# Modeling Gene-Environment Interaction for the Risk of Non-hodgkin Lymphoma

**DOI:** 10.3389/fonc.2018.00657

**Published:** 2019-01-14

**Authors:** Jiahui Zhang, Xibiao Ye, Cuie Wu, Hua Fu, Wei Xu, Pingzhao Hu

**Affiliations:** ^1^Division of Biostatistics, Dalla Lana School of Public Health, University of Toronto, Toronto, ON, Canada; ^2^Department of Community Health Science, Rady Faculty of Health Sciences, Max Rady College of Medicine, University of Manitoba, Winnipeg, MB, Canada; ^3^School of Public Health, Fudan University, Shanghai, China; ^4^Department of Biostatistics, Princess Margaret Cancer Centre, Toronto, ON, Canada; ^5^Department of Biochemistry and Medical Genetics, Faculty of Health Sciences, College of Medicine, University of Manitoba, Winnipeg, MB, Canada; ^6^Research Institute in Oncology and Hematology, Winnipeg, MB, Canada

**Keywords:** gene-environment interaction (G × E), genetic risk score (GRS), clustering (unsupervised) algorithms, non-hodgkin lymphoma, candidate genes

## Abstract

**Background:** Non-hodgkin lymphoma (NHL) is one of the most common and deadly cancers. There is limited analysis of gene-environment interactions for the risk of NHL. This study intends to explore the interactions between genetic variants and environmental factors, and how they contribute to NHL risk.

**Methods:** A case-control study was performed in Shanghai, China. The cases were diagnosed between 2003 and 2008 with patients aged 18 years or older. Samples and SNPs which did not satisfy quality control were excluded from the analysis. Weighted and unweighted genetic risk scores (GRS) and environmental risk scores were generated using clustering analysis algorithm. Univariate and multivariable logistic regression analyses were conducted. Moreover, genetics and environment interactions (G × E) were tested on the NHL cases and controls.

**Results:** After quality control, there are 22 SNPs, 11 environmental variables and 5 demographical variables to be explored. For logistic regression analyses, 5 SNPs (rs1800893, rs4251961, rs1800630, rs13306698, rs1799931) and environmental tobacco smoking showed statistically significant associations with the risk of NHL. Odds ratio (OR) and 95% confidence interval (CI) was 10.82 (4.34–28.88) for rs13306698, 2.84 (1.66–4.95) for rs1800893, and 2.54 (1.43–4.58) for rs4251961. For G × E analysis, the interaction between smoking and dichotomized weighted GRS showed statistically significant association with NHL (OR = 0.23, 95% CI = [0.09, 0.61]).

**Conclusions:** Several genetic and environmental risk factors and their interactions associated with the risk of NHL have been identified. Replication in other cohorts is needed to validate the results.

## Introduction

NHL is the eighth most frequent type of cancers in men and the eleventh in women worldwide (1). American Cancer Society estimates that 74,680 people will be diagnosed with NHL, and 19,910 NHL deaths will occur in 2018 (2). Canadian Cancer Society estimates that 8,300 people will be diagnosed with NHL, and 2,700 will die from the disease (3). Studies showed that the overall incidence rate has increased for both males and females from 1973 to 2010 in Shanghai, China (4, 5).

NHL occurs more often in males than in females. It is most frequently diagnosed in the 65–74 age group. The known risk factors for developing NHL include weakened immune system, previous cancer treatment; such as radiation therapy and chemotherapy, hepatitis C virus (HCV) and hepatitis B virus (HBV) infection (6). Some possible causes of NHL disease might include chemical exposure and medical treatments (7). Environmental risk factors, such as experience of benzene exposure that exceeds 810 days, daily welding and activity of radio operator, were associated with NHL from a previous case- control study taken place in France (8).

Genetic factors were also associated with the risk of NHL. For example, a hospital-based study conducted in China showed that rs1800893 in IL-10, rs4251961 in IL-1RN, rs1800630 in TNF- a and rs2229094 in TNF- a had association with an increased risk of overall NHL (4). Another genome-wide association study (GWAS) in a Chinese population showed two SNPs (rs872071 in IRF4 and rs2647012 in HLA class II) were associated with increased risk of NHL (9). Previous studies found that environmental factors and genetic variants interaction may affect risk of complex diseases (10, 11). A U.S. study suggested that usage of hair dye before 1980 increased the risk of NHL due to genetic variation in NAT1 and NAT2 genes (12). A G × E interaction analysis conducted by Gathany et al. suggested that sun exposure and sensitivity increased risk of NHL, which is interceded by IRF 4 (13). A more subtype specific study suggested several statistically significant interaction terms between risk factors and gene (14). This study focused on statistical analysis of genetic and environmental factors and their interactions with the risk of NHL. Previous research of NHL had examined the association between genetic variants in some candidate genes and the risk of NHL in Shanghai, China [e.g., (4)]. We conducted this study to gain new insight into the G × E effect on the risk of NHL.

## Method

**Figure 1:**
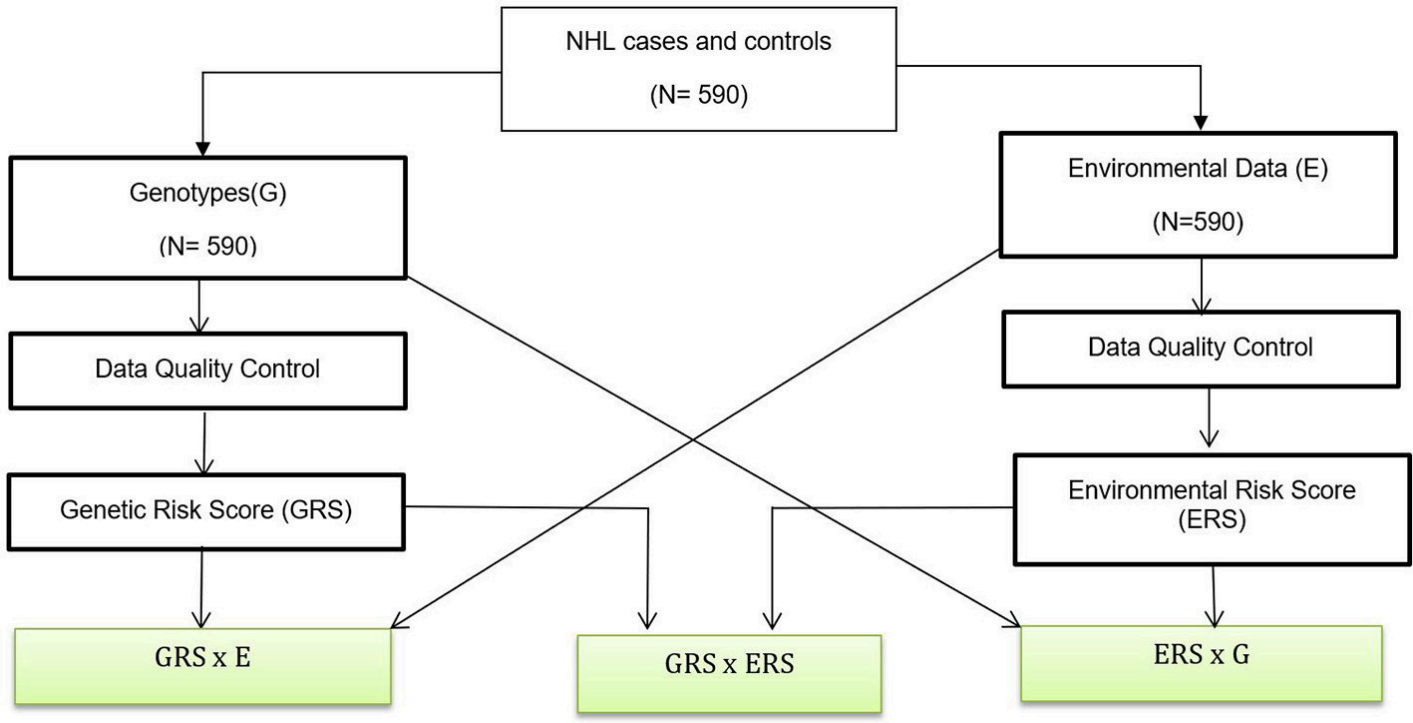
Data Analysis Flow. A genetic association analysis with 590 NHL and control participants and their biological samples were gathered. Data quality control are performed for both genotypes and environmental variables. We also created GRSs and ERS for interaction analysis. Boxes with green fill are interaction analysis.

The data analysis flow is shown in Figure 1. Below we briefly summarized each of the analysis steps.

### Study Samples

The participants in this study were from Shanghai, China. This study included 169 NHL cases and 421 controls. NHL patients in this longitudinal population-based study were diagnosed in a span of 5 years (from 2003 to 2008) and were aged 18 years or older. Controls were randomly recruited from the same hospital in Shanghai, China, and matched by age and gender group frequency of cases (4). Those controls with cancers or non-malignant lymphatic or hematopoietic diseases, or having family connections to the samples were excluded. Each participant completed a questionnaire containing various questions which formed the environmental variables used in the study. Peripheral blood, bone marrow aspirates, tissue and core biopsies of the subjects were collected and sent to the laboratory for analyses. RFLP (Restriction Fragment Length Polymorphism) was used for DNA genotyping of the blood samples on an ABI 7900HT sequence detection system at the State Key Laboratory of Genetics in Fudan University, China. This study has been approved by the Ethical Review Committee of Fudan University in China. Informed consent was obtained from all individual participants in the study.

### Environmental and Genetic Data Quality Control

Quality control analysis was performed for environmental and genetic variables, which are detailed below. PLINK tool was used to perform genetic data quality control and genetic association analysis (15). R programming tool was used to perform all the environmental and other demographical and epidemiological data quality control and other association analyses (16).

#### Hardy-Weinberg Equilibrium (HWE) Check

The deviation of HWE can indicate genotyping errors. HWE test had been applied to the control group (17). The *p*-value threshold was 0.0001, which means the SNPs with *p*-value of HWE test <0.0001 were excluded for the follow-up analysis.

#### SNP Missing Rate Check

SNP missing rate is defined as the number of missing genotypes divided by the total number of participants in the study. SNPs with high missing rate might cause higher false positive rate. Therefore, we removed SNPs with missing rate higher than 0.1 from the study.

#### Environment Data Quality Control

For environmental variables, we mainly focused on 21 variables that are potentially relevant to NHL (4). In this analysis, environmental variables with missing rate higher than 0.05 were excluded. For categorical variables, we used chi-square test to evaluate the significance of the association between each variable and NHL outcomes. For continuous variables, we used *t*-test and Wilcoxon test to assess the significance of the association between each variable and NHL outcomes.

### Statistical Methods

We summarized environmental variables for all samples, case group and control group. For continuous variables; mean, standard deviation, median, range, and missing number were displayed. For categorical variables, frequency and proportion were presented. When calculating proportion of each variable, we did not include missing number (Table 1). Two-sided test was used and significance level was set as 0.05. Benjamini and Hochberg (BH) procedure was applied to adjust for multiple comparisons (18). Logistic regression analysis was implied to calculate OR and corresponding 95% confidence interval (19).

**Table 1:** Characteristics of NHL Cases and Controls.

**Study Variables**	**Full Sample (*n* = 590)**	**Controls (*n* = 421)**	**Cases (*n* = 169)**
**NON-ENVIRONMENTAL VARIABLES**			
**Age**^***a***^			
Mean (sd^*b*^)	57.1 (13.5)	57.9 (13.7)	55.1 (12.9)
Median (Min,Max^*c*^)	56 (12,90)	57 (12,90)	55 (19,86)
Missing	4	4	0
**Gender**			
female	361 (61)^*d*^	258 (61)	103 (61)
male	229 (39)	163 (39)	66 (39)
**Education**^***a***^			
none/prime school	217 (37)	73 (17)	144 (85)
middle/high school or higher	373 (63)	348 (83)	25 (15)
**Family History of Cancer**^***a***^			
No	373 (63)	322 (77)	51 (30)
Yes	216 (37)	98 (23)	118 (70)
Missing	1	1	0
**Bmi**^***a***^			
Mean (sd^*b*^)	23.9 (6)	24.3 (6.8)	22.9 (3.1)
Median (Min,Max^*c*^)	23.4 (15.1,142.4)	24 (16.4,142.4)	22.8 (15.1,33.1)
Missing	3	3	0
**ENVIRONMENTAL VARIABLES**			
**Smoking**			
No	361 (61)	267 (64)	94 (56)
Yes	228 (39)	153 (36)	75 (44)
Missing	1	1	0
**Alcohol**			
No	456 (78)	328 (78)	128 (76)
Yes	132 (22)	91 (22)	41 (24)
Missing	2	2	0
**Hairdye**			
No	384 (65)	279 (66)	105 (62)
Yes	205 (35)	142 (34)	63 (38)
Missing	1	0	1
**Lived on Farm**^***a***^			
No	381 (65)	301 (71)	80 (48)
Yes	208 (35)	120 (29)	88 (52)
Missing	1	0	1
**Environmental Tobacco Smoking**^***a***^			
No	336 (57)	211 (50)	125 (74)
Yes	254 (43)	210 (50)	44 (26)
**Benzene Exposure**^***a***^			
No	579 (98)	421 (100)	158 (93)
Yes	11 (2)	0 (0)	11 (7)
**Solvent Exposure**			
No	509 (86)	361 (86)	148 (88)
Yes	81 (14)	60 (14)	21 (12)
**Metal Exposure**			
No	568 (96)	407 (97)	161 (95)
Yes	22 (4)	14 (3)	8 (5)
**Agricultural Chemical Exposure**^***a***^			
No	518 (88)	395 (94)	123 (73)
Yes	72 (12)	26 (6)	46 (27)
**Other Occupational Exposures**^***a***^			
No	549 (93)	385 (91)	164 (97)
Yes	41 (7)	36 (9)	5 (3)
**Pesticide Exposure**^***a***^			
No	522 (88)	396 (94)	126 (75)
Yes	68 (12)	25 (6)	43 (25)

a *Variables have p-value with chi-square test or t-test <0.05*.

b Sd refers to standard deviation;

c Min refers to the minimum value and Max refers to the maximum value in each group (Full Samples, Control, and Cases) for continuous variables.

d *The number in bracket is proportion (%) of the non-missing number in each group. For proportion calculation in brackets for category variables, we only used observed data*.

#### Univariate and Multivariable Logistic Regression Analyses

For the univariate SNP-based association analysis, we coded genotypes into numeric values using an additive genetic model (20). We applied the Cochran-Armitage trend test and then adjusted *p*-values using BH multiple testing (21).

To evaluate the association between NHL risk and each environmental variable, univariate logistic regression model was first performed (20).

(1) $$\begin{array}{r} \text{logit }\left(\text{NHL}\right)={\beta_{0}+\beta }_{x}\;*\;X \end{array}$$

where β_0_ represented the intercept term, β_*x*_ referred to the effect of each environmental variable, *X* was each environmental variable.

Multivariable logistic regression model was also implemented for each environmental variable or SNP by adjusting age, gender, education, family history of cancer, and body mass index as a prior (4).

(2) $$\begin{array}{l} \text{logit }(\text{NHL})=\beta_{0}+\beta_{x}*X+\beta_{1}*age+\beta_{2}*\text{ gender }+\beta_{3}*\text{edu}\\ \;+\beta_{4}*\text{fh}+\beta_{5}*\text{BMI} \end{array}$$

where β_1_, β_2_, β_3_, β_4_, β_5_ were regression coefficient for age, gender, education, family history (fh) of NHL and BMI; *X* was each environmental variable or SNP. *P*-values of β_*x*_ were adjusted using BH method.

#### Interaction Analysis Between Environment Risk Score With SNPs

Following the idea of Park et al. (22), we generated an environmental risk score (ERS) as a new variable using the clustering method implemented in blockcluster R package (23). Block Clustering is a data mining technique. It groups samples into different clusters. Samples in the same group/cluster are more similar than in other groups. In this analysis, we focused on the semi-supervised coclusterBinary algorithm which analyzes the binary variables and simultaneously clusters both variables and study samples. It applied conditional expectation maximization (CEM) to estimate the unknown parameters. Since it is a semi-supervised clustering approach, a label is needed to be assigned to each binary environmental variable. To do this, we coded each given environmental variable with label 1 if its odds ratio is larger than 1 in the logistic regression analysis based on Formula (1). Otherwise, it is coded as 0. The clustering procedure will assign each of the environmental variables into environmental risk group or non- environmental risk group.

We performed the interaction analysis between the ERS with each of the SNPs with and without adjusting age, gender, education, family history of NHL, and body mass index (BMI) (24) as shown in Formula (3):

(3) $$\begin{array}{l} \text{logit }(\text{NHL})=\beta_{0}+\beta_{ERS}*ERS+\beta_{snp}*SNP+\beta_{int}*\text{ERS}*\text{SNP}\\ \;+\beta_{1}*age+\beta_{2}*\text{ gender }+\beta_{3}*\text{edu}+\beta_{4}*\text{fh}\\ \;+\beta_{5}*\text{BMI} \end{array}$$

β_0_ represented the intercept term; β_*ERS*_, β_*snp*_ referred to the regression coefficients for ERS and each SNP, respectively; β_1_, β_2_, β_3_, β_4_, β_5_ were regression coefficient for age, gender, education, family history of NHL and BMI; β_*int*_ was the regression coefficient for the interaction term of ERS and SNP; *P*-values of β_*int*_ were adjusted using BH multiple testing method.

#### Interaction Analysis Between Environmental Variables With GRS

We generated weighted and unweighted genetic risk scores (GRS) based on the 22 SNPs, respectively. We used the “riskScore” function in PredictABEL R package to calculate the GRS (25). Briefly speaking, a univariate logistic regression model was fitted for each SNP. Unweighted GRS (U_GRS) of a given sample was the sum of risk alleles across all 22 SNPs. Weighted GRS (W_GRS) of a given sample was the number of risk alleles rescaled by its relative effect size (regression coefficient in the logistic regression model). We dichotomized these two GRSs (W_GRS and U_GRS) based on their median. If a sample had a GRS greater than the median of GRS, we assigned 1 to the sample. Otherwise, we assigned 0 to the sample.

We performed interaction analysis between the dichotomized GRSs with each of the environmental variables with and without adjusting age, gender, education, family history of NHL and body mass index; as shown in Formula (4):

(4) $$\begin{array}{l} \text{logit }(\text{NHL})=\beta_{0}+\beta_{GRS}*\text{GRS}+\beta_{E}*E+\beta_{\text{int}}*\text{GRS}*\text{E}+\beta_{1}*\text{ age}\\ \;+\beta_{2}*\text{ gender }+\beta_{3}*\text{edu}+\beta_{4}*fh+\beta_{5}*\text{BMI} \end{array}$$

where β_*GRS*_ referred to the regression coefficient for dichotomized unweighted/weighted GRS; *E* was an environment variable; β_*int*_ was the regression coefficient for the interaction term of GRS and environmental variable (E). *P*-values of β_*int*_ were adjusted using BH method.

## Results

### Data Quality Control and Characteristics of Variables

In this study, we focused on 21 environmental and non-environmental variables from our previous study (4). Five variables, including smok100 (smoked more than 100 cigarettes), NOEH3 (age started smoking), smok_num (Average smoked number of cigarettes per day), voltage exposure and radiation exposure were removed from the study as they exhibited a missing rate larger than 0.05. A total of sixteen variables were left for follow-up analysis. These included 5 non-environmental variables and 11 environmental variables. Table 1 shows the comparison of characteristics of these variables in this study. Smoking status, drink alcohol, and dye hair were similarly distributed in cases and controls. Cases were less educated and more prone to environmental and occupational exposures, such as living on farm, experience of environmental tobacco smoking, benzene exposure, agricultural chemical exposure, and pesticide exposure (4). Family history of cancer was significantly associated with risk of NHL in this study. Therefore, the five non-environmental variables [sex, age, education, family history of cancer, and body mass index (BMI)], named as covariates, were adjusted in our multivariable logistic regression analysis.

**Figure 2:**
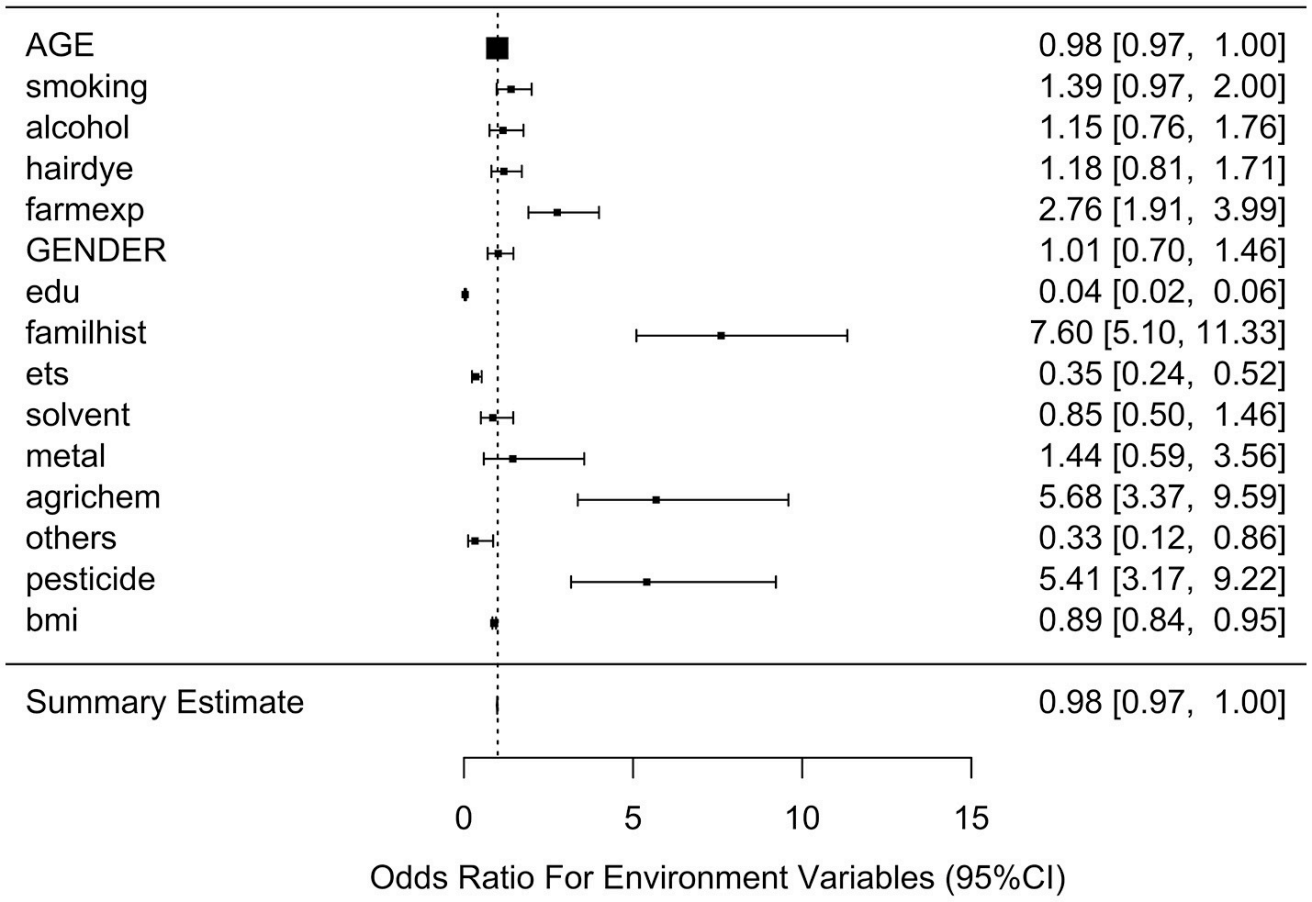
Odds ratios (95% confidence intervals) of risk of NHL for each environmental variable.

Table 2 lists the result of logistic regression analyses with and without adjusting the five covariates for each of the 11 environmental variables. For univariate analysis, five environmental variables (living on farm, environmental tobacco smoking, agricultural chemical exposure, other occupational exposure and pesticide exposure), showed significant association with NHL. Four (living on farm, environmental tobacco smoking, agricultural chemical exposure, and pesticide exposure) of the five environmental variables showed statistically significance after adjusting multiple testing by BH method. For multivariable logistic analysis, three environmental variables (hair dying, environmental tobacco smoking and pesticide exposure) showed statistically significance with risk of NHL. Two (hair dying and environmental tobacco smoking) of the three environmental variables from multivariable logistic analysis presented significant *p*-values after multiple testing adjustment by BH method. Figure 2 shows the odds ratio and 95% confidence interval for each environmental variable with the risk of NHL. Agricultural chemical exposure (OR = 5.68, 95% CI = 3.4–9.69) and pesticide exposure (OR = 5.41, 95%CI = 3.2–9.31) were associated with an increased risk of NHL.

**Table 2:** Associations between environmental variables and NHL risk.

**Environmental Variable**	**OR (95% CI)^***a***^**	**P_u_^*b*^**	**P_m_*^c^***	**P_u_BH_^*d*^**	**P_m_BH_^*e*^**
Smoking	1.39 (0.97, 2)	0.07	0.12	0.14	0.19
Alcohol	1.15 (0.75, 1.75)	0.50	0.28	0.62	0.35
*Hairdye*	**1.18 (0.81, 1.71)**	**0.39**	**0.002**	**0.57**	**0.01**
Lived on farm	**2.76 (1.91, 4)**	**7.24E-08**	**0.93**	**2.65E-07**	**0.98**
*Environmental tobacco smoking*	**0.35 (0.24, 0.52)**	**2.19E-07**	**0.0002**	**6.03E-07**	**0.002**
Benzene	NA (0, NA)	0.97	0.98	0.97	0.98
Solvent	0.85 (0.49, 1.43)	0.56	0.05	0.62	0.13
Metal	1.44 (0.57, 3.44)	0.42	0.20	0.57	0.27
Agrichem	**5.68 (3.4, 9.69)**	**6.75E-11**	**0.07**	**7.43E-10**	**0.13**
Others	**0.33 (0.11, 0.77)**	**0.02**	**0.06**	**0.05**	**0.13**
Pesticide	**5.41 (3.2, 9.31)**	**5.13E-10**	**0.04**	**2.82E-09**	**0.13**

a OR, odds ratio; 95% CI, 95% confidence interval.

b P_U_ is the p value of univariate analysis with NHL.

c P_m_ is the p value using multivariable logistic model adjusted for age, gender, education, family history of cancer and BMI.

d *P_U_BH_ and ^e^P_m_BH_ are P_U_ and P_m_ adjusted by BH method. Bolded variables are statistically significant in either univariate or multiple logistic analysis adjusted by BH method*.

### SNP Data Quality Control and Association Analysis

There are 29 SNPs genotyped for the study. Three SNPs, rs1051740, rs1346044, and rs2227973 were removed from the further analysis as they presented a Hardy-Weinberg Equilibrium (HWE) test *P*-value <0.0001 in control group. Four SNPs rs2229094, rs1799930, rs915906, and rs13181 were removed from the analysis as their missing rates were higher than 0.1.

**Figure 3:**
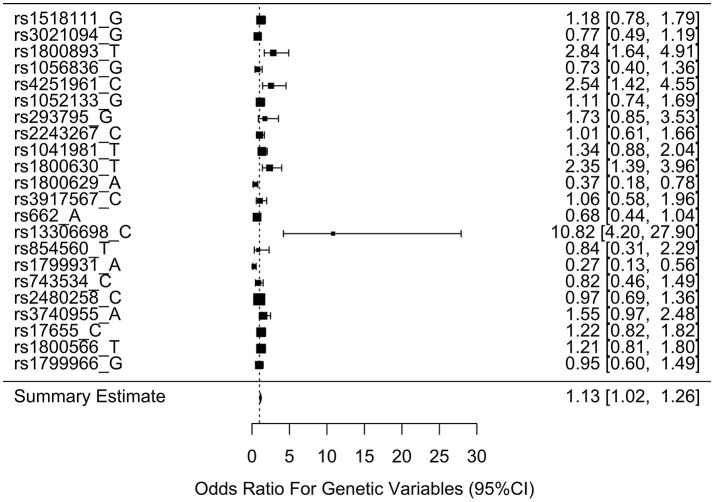
Odds ratios (95% confidence intervals) of risk of NHL for each SNP. This is based on the results from Table 3.

Table 3 shows the associations between gene SNPs and NHL risk. Five SNPs (rs1800893, rs4251961, rs1800630, rs13306698, and rs1799931) showed statistically significance in both univariate and multivariable logistic analyses after adjusting multiple testing by BH method. Table 3 and Figure 3 show the odds ratio and 95% confidence interval for each SNP. rs13306698 featured the highest odds ratio and 95% confidence interval (OR = 10.82, 95% CI = 4.34–28.88); it was associated with an increased risk of NHL. rs1800893 (OR = 2.84, 95% CI = 1.66–4.95) and rs4251961(OR = 2.54, 95% CI = 1.43–4.58) also showed that they have significant association with an increased risk of NHL.

**Table 3:** Associations between SNPs and NHL risk.

**Gene**	**SNP**	**Minor allele**	**Alternative allele**	**UNAFF Count^***a***^**	**AFF Count^***b***^**	**OR (95% CI) ^***c***^**	**P_ca_BH^*d*^_**	**P_m_BH^*e*^_**
IL-10	rs1518111	G	A	52/193/150	15/89/58	1.18 (0.78, 1.79)	0.78	0.65
IL-10	rs3021094	G	T	72/208/103	24/90/41	0.77 (0.49, 1.19)	0.73	0.48
IL-10	**rs1800893**	**T**	**C**	**5/125/260**	**3/90/68**	**2.84** (**1.66, 4.95)**	**3.00E-06**	**0.002**
CYP1B1	rs1056836	G	C	8/109/267	1/41/113	0.74 (0.39, 1.34)	0.62	0.55
IL-1RN	**rs4251961**	**C**	**T**	**4/73/311**	**7/46/109**	**2.54 (1.43, 4.58)**	**0.002**	**0.007**
OGG1	rs1052133	G	C	89/186/123	31/99/32	1.11 (0.74, 1.69)	0.49	0.79
OGG1	**rs293795**	**G**	**A**	**1/30/386**	**2/22/145**	**1.73 (0.85, 3.53)**	**0.03**	**0.31**
IL-4	rs2243267	C	G	14/180/188	6/75/74	1.01 (0.61, 1.66)	0.78	0.98
TNF	rs1041981	T	G	72/207/105	32/93/30	1.34 (0.88, 2.05)	0.32	0.39
TNF	**rs1800630**	**T**	**G**	**16/101/270**	**2/77/83**	**2.35 (1.4, 3.98)**	**0.01**	**0.007**
TNF	**rs1800629**	**A**	**G**	**2/61/319**	**1/20/134**	**0.37 (0.17, 0.76)**	**0.73**	**0.03**
PON1	rs3917567	C	T	4/74/317	4/29/129	1.06 (0.57, 1.96)	0.73	0.89
PON1	rs662	A	G	51/191/142	20/74/61	0.68 (0.44, 1.03)	0.73	0.20
PON1	**rs13306698**	**C**	**T**	**0/18/379**	**0/46/116**	**10.82 (4.34, 28.88)**	**2.01E-14**	**2.00E-05**
PON1	rs854560	T	A	2/26/356	0/15/140	0.84 (0.3, 2.23)	0.73	0.89
NAT2	**rs1799931**	**A**	**G**	**6/90/288**	**1/22/139**	**0.27 (0.13, 0.54)**	**0.02**	**0.003**
CYP2E1	rs743534	C	A	12/109/262	2/37/116	0.82 (0.45, 1.48)	0.30	0.72
CYP2E1	rs2480258	C	T	104/166/129	44/57/64	0.97 (0.69, 1.36)	0.73	0.89
RAG1	rs3740955	A	G	16/141/255	8/67/92	1.55 (0.97, 2.48)	0.35	0.20
ERCC5	rs17655	C	G	89/191/104	40/74/41	1.22 (0.82, 1.82)	0.73	0.55
NQO1	rs1800566	T	C	80/197/107	39/72/44	1.21 (0.81, 1.8)	0.73	0.55
BRCA1	rs1799966	G	A	45/194/156	14/95/53	0.95 (0.6, 1.49)	0.73	0.89

a UNAFF is the count of genotypes (rare homozygosity/ heterozygosity /common homozygosity) in controls;

b AFF is the count of genotypes in cases.

c OR, odds ratio; 95% CI, 95% confidence interval.

d P_ca_BH_ is the p-value of Cochran-Armitage trend test adjusted by BH method.

e *P_m_BH_ is the p-value adjusted by BH method using multivariable logistic model adjusted for age, gender, education, family history of cancer and BMI. Bolded variables are statistically significant adjusted by BH method in either univariate or multiple logistic analysis*.

### Gene and Environment Interaction Analysis

**Figure 4:**
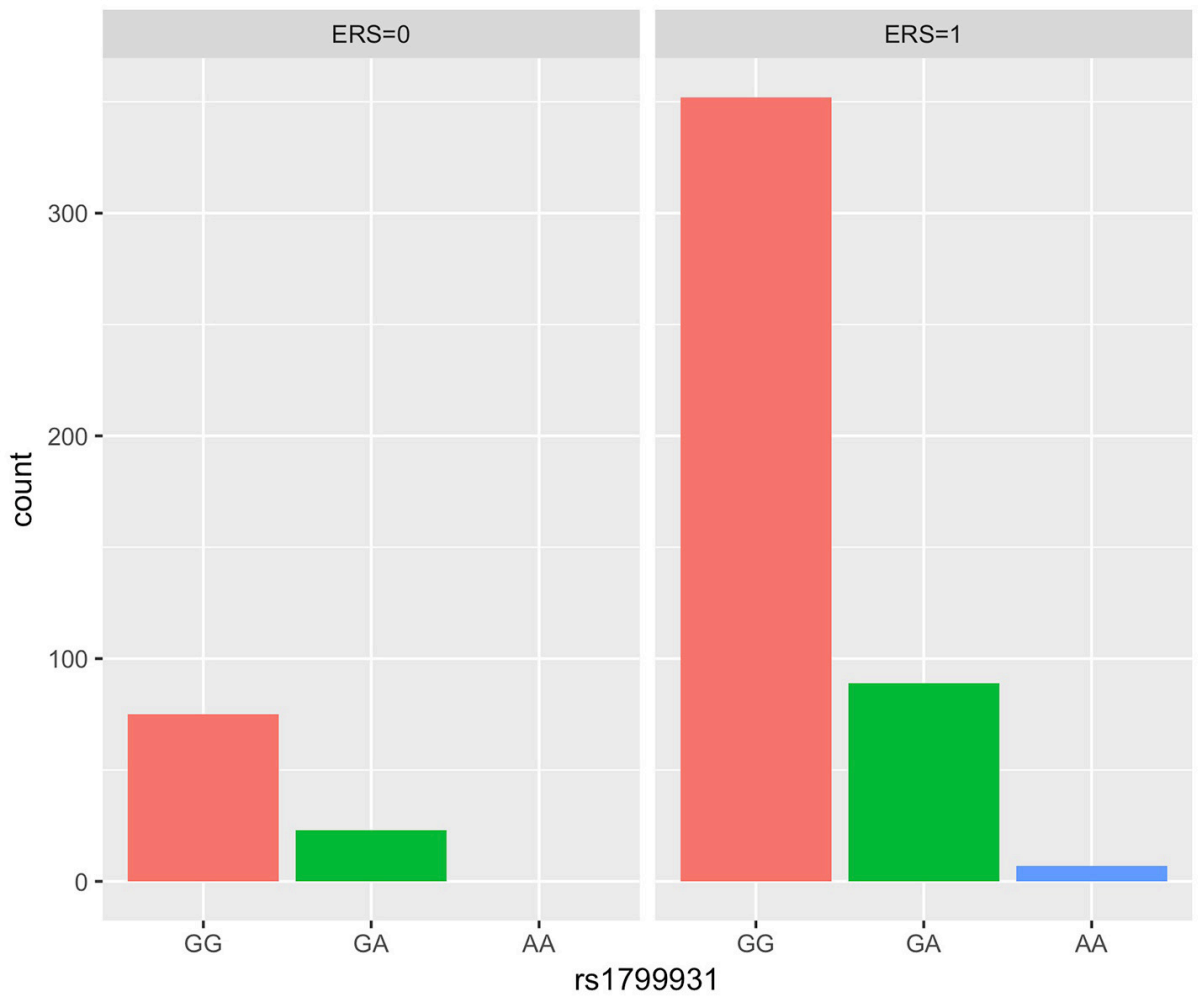
Frequency of genotype plot of rs1799931 in environmental risk group (ERS = 1) and non-environmental risk group (ERS = 0). Frequency of genotypes GG is shown in red, GA is shown in green and AA is shown in blue.

We generated ERS using the block cluster method. Table 4 shows the interaction analysis between the ERS and each SNP. rs1799931 showed significant nominal *p*-value for interaction analysis after adjusting the five covariates, but no significant *p*-values after adjusting multiple testing. For the most significant SNP rs1799931 and interaction with ERS, Figure 4 shows the frequencies of its genotypes (rare homozygosity/ heterozygosity /common homozygosity) in the environmental risk group (ERS = 1) compared with the non-environmental risk group (ERS = 0). As shown in the figure, the frequencies of the common homozygosity GG in the environmental risk group was much higher than frequencies in the non- environmental risk group.

**Table 4:** Interaction analysis between the environmental risk score variable and each SNP.

**SNP**	**ROR (95% CI) ^***a***^**	**P^***b***^**	**P_BH_*c***	ROR*(95% CI*) ^***d***^	P*^e^	P_BH_^*f^
rs1518111	1.39 (0.7, 2.76)	0.35	0.91	2.83 (0.97, 8.28)	0.06	0.64
rs3021094	1.18 (0.58, 2.4)	0.64	0.91	0.7 (0.21, 2.28)	0.55	0.87
rs1800893	1.29 (0.54, 3.06)	0.56	0.91	2.96 (0.82, 10.64)	0.10	0.70
rs1056836	1.32 (0.5, 3.46)	0.57	0.91	0.84 (0.19, 3.72)	0.81	0.87
rs4251961	0.95 (0.35, 2.57)	0.92	0.96	1.39 (0.3, 6.46)	0.68	0.87
rs1052133	1.4 (0.74, 2.64)	0.30	0.91	1.56 (0.56, 4.34)	0.39	0.87
rs293795	1.37 (0.38, 4.94)	0.63	0.91	0.48 (0.07, 3.16)	0.45	0.87
rs2243267	1.18 (0.54, 2.58)	0.68	0.91	1.6 (0.45, 5.7)	0.47	0.87
rs1041981	1 (0.5, 2.01)	0.99	0.99	0.77 (0.25, 2.37)	0.64	0.87
rs1800630	0.63 (0.27, 1.49)	0.29	0.91	1.13 (0.26, 4.87)	0.87	0.87
rs1800629	1.68 (0.49, 5.71)	0.41	0.91	1.66 (0.25, 11.06)	0.60	0.87
rs3917567	0.49 (0.18, 1.39)	0.18	0.91	0.56 (0.11, 2.95)	0.49	0.87
rs662	1.29 (0.66, 2.54)	0.46	0.91	1.6 (0.56, 4.63)	0.38	0.87
rs13306698	0.75 (0.14, 3.99)	0.73	0.91	0.54 (0.02, 18.05)	0.73	0.87
rs854560	4.61 (0.85, 24.93)	0.08	0.91	3.56 (0.19, 67.4)	0.40	0.87
rs1799931	0.54 (0.18, 1.63)	0.27	0.91	0.06 (0.01, 0.44)	0.006	0.12
rs743534	0.45 (0.18, 1.13)	0.09	0.91	0.55 (0.12, 2.52)	0.44	0.87
rs2480258	1.5 (0.84, 2.71)	0.17	0.91	0.91 (0.37, 2.26)	0.84	0.87
rs3740955	1.19 (0.59, 2.4)	0.62	0.91	1.99 (0.67, 5.89)	0.21	0.87
rs17655	0.9 (0.46, 1.76)	0.76	0.91	0.7 (0.23, 2.13)	0.53	0.87
rs1800566	1.05 (0.54, 2.02)	0.89	0.96	0.57 (0.19, 1.67)	0.31	0.87
rs1799966	1.1 (0.54, 2.25)	0.79	0.91	0.85 (0.26, 2.83)	0.79	0.87

a ROR, rate of odds ratio; 95% CI, 95% confidence interval.

b P, p-value of interaction analysis between ERS and SNP.

c P_BH_ is the p-value adjusted by BH method.

d ROR*, rate of odds ratio after adjusting age, gender, education, family history of cancer and BMI; 95% CI*, 95% confidence interval after adjusting age, gender, education, family history of cancer and BMI.

e P*, p-value of interactive term using multivariable logistic model with adjusting age, gender, education, family history of cancer and BMI.

f P_BH_* is the p* value adjusted by BH method.

Supplementary Figures 1, 2 show rates of odds ratio with and without adjusting the 5 covariates. With the five covariates adjusted in the logistic regression analysis, the interaction of ERS and rs1799931 presented the lowest rate of odds ratio (ROR = 0.06, 95% CI = 0.01–0.44); it was associated with a decrease risk of NHL. As observed from these two figures, the interaction of ERS and rs854560 yielded the highest ratio of odds ratio (ROR = 4.61, 95% CI = 0.85–24.93; ROR^*^ = 3.56, 95% CI^*^ = 0.19–67.4); it was associated with an increased risk of NHL.

**Figure 5:**
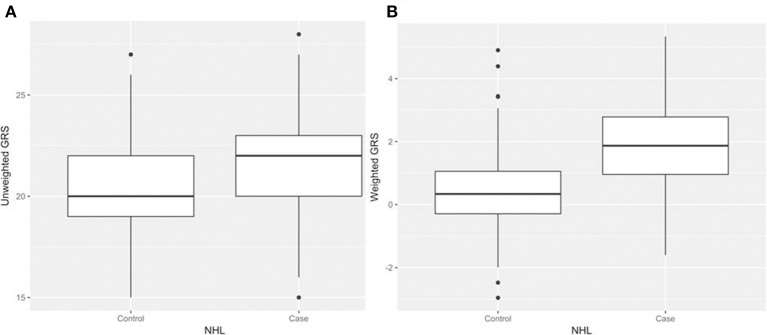
Boxplots of genetic risk scores (GRSs) in control and case groups respectively. **(A)** unweighted GRSs **(B)** and weighted GRSs.

**Figure 6:**
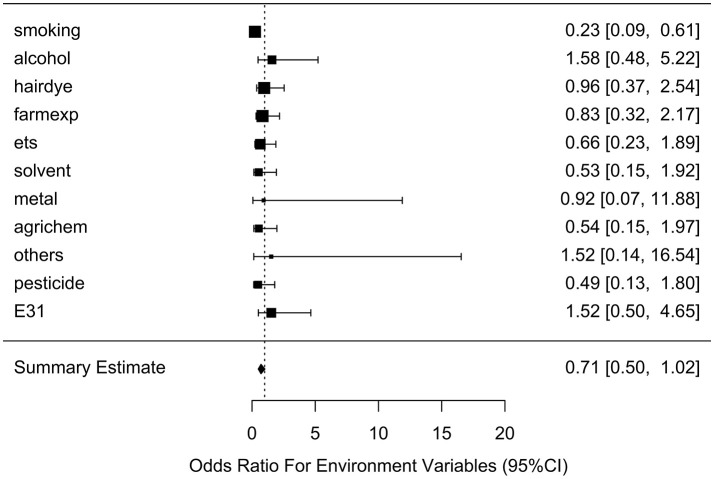
Ratio of Odds ratios (95% confidence intervals) of risk of NHL based on unadjusted models. This is based on the results from Table 5. The environmental variable benzene has not been included due big odds ratio. Models are not adjusted for age, gender, education, family history of cancer and BMI. E31 is environmental risk score (ERS).

**Figure 7:**
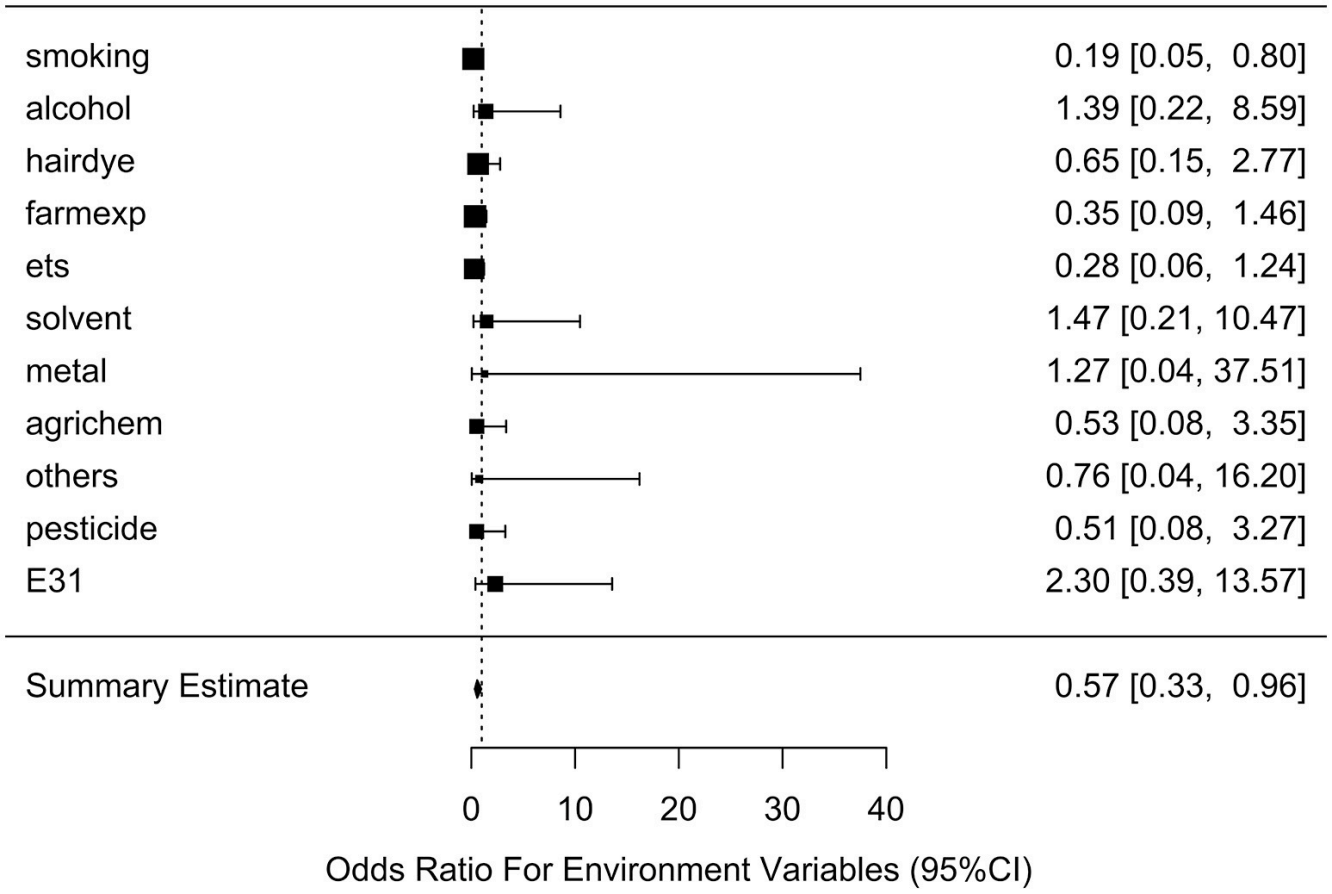
Ratio of Odds ratios (95% confidence intervals) of risk of NHL based on adjusted models. This is based on the results from Table 5. The environmental variable benzene has not been included due big odds ratio. Models are adjusted for age, gender, education, family history of cancer and BMI. E31 is environmental risk score (ERS).

After generating the environmental risk variable, we generated weighted and unweighted GRSs. Figure 5 shows the distributions of the two GRSs comparing the case and control groups. The median of unweighted GRS was much larger than the weighted GRS. For both GRSs, median and quantiles in the case group was higher than those in the control group. Table 5 shows the interaction analysis between individual environmental variable and the dichotomized weighted GRS. The interaction of dichotomized weighted GRS and smoking showed significant nominal *p*-value for both with and without adjusting the five covariates (*P* = 0.003; P^*^ = 0.02). After adjusting for multiple testing, the interaction of GRS and smoking still showed significant association with NHL (*P*_*BH*_ = 0.035). Figures 6, 7 show rates of odds ratio with and without adjusting the 5 covariates. The interaction of GRS and smoking presents the lowest rate of odds ratio (ROR = 0.23, 95% CI = 0.09–0.61).

**Table 5:** Interaction analysis between each environmental variable and weighted GRS.

**Environmental Variable**	**ROR(95% CI) ^***a***^**	**P^***b***^**	**P_BH_*c***	ROR*(95% CI*) ^***d***^	P*^e^	P_BH_*^f^
Smoking	**0.23** (**0.09, 0.61)**	**0.003**	**0.035**	**0.19** (**0.05, 0.8)**	**0.02**	**0.29**
Alcohol	1.58 (0.48, 5.22)	0.45	0.79	1.39 (0.22, 8.59)	0.73	0.97
Hairdye	0.96 (0.37, 2.54)	0.94	1	0.65 (0.15, 2.77)	0.56	0.96
Farmexp	0.83 (0.32, 2.17)	0.7	0.98	0.35 (0.09, 1.46)	0.15	0.60
Environmental tobacco smoking	0.66 (0.23, 1.89)	0.43	0.79	0.28 (0.06, 1.24)	0.09	0.56
Benzene	0.13 (0, Inf)	1	1	0.17 (0, Inf)	1	1
Solvent	0.53 (0.15, 1.92)	0.33	0.79	1.47 (0.21, 10.47)	0.7	0.97
Metal	0.92 (0.07, 11.88)	0.95	1	1.27 (0.04, 37.51)	0.89	0.97
Agrichem	0.54 (0.15, 1.97)	0.35	0.79	0.53 (0.08, 3.35)	0.5	0.96
Others	1.52 (0.14, 16.54)	0.73	0.98	0.76 (0.04, 16.2)	0.86	0.97
Pesticide	0.49 (0.13, 1.8)	0.28	0.79	0.51 (0.08, 3.27)	0.48	0.96

a ROR, rate of odds ratio; 95% CI, 95% confidence interval.

b P, p-value of interaction analysis between dichotomized weighted GRS and environmental variables.

c P_BH_ is the p-value adjusted by BH method.

d ROR*, rate of odds ratio after adjusting age, gender, education, family history of cancer and BMI.; 95% CI*, 95% confidence interval after adjusting age, gender, education, family history of cancer and BMI.

e P*, p-value of interactive term using multiple logistic model with adjusting age, gender, education, family history of cancer and BMI.

f P_BH_* is the p^*^ value adjusted by BH method. The bolded interaction term is statistically significant.

Interaction analysis result between individual environmental variable and the dichotomized weighted GRS is shown in Supplement Table 1. GRS and ERS interaction analysis result is shown in Supplement Table 2. Both do not have statistical significance.

## Discussion

Our previous study has shown that SNPs in immune-regulatory genes (*IL-10* and *TNF*) are significantly associated with the risk of NHL (4) in a Chinese population in Shanghai. The current study mainly focused on the effect of gene and environment interactions on the risk of NHL. For the gene and environment interaction analysis without adjusting the five covariates, we observed that the interaction of smoking and weighted GRS has statistically significant association with NHL. However, smoking did not show directly significant association with the risk of NHL in univariate or multivariable logistic models. We did not observe other environmental variables that interacted with GRS to affect the risk of NHL.

NHL is a cancer of immune system. It is well-known that immune deficiency is one of a few well-established risk factors for NHL. For example, polymorphisms in the promoter region of the *TNF* gene and the *IL-10* were reported to be associated with increased NHL risk, and particularly increased DLBCL risk (4, 26–29). However, the specific immune mechanisms responsible remain unresolved. As we know, immunologic response is often driven by specific environmental agents that are influenced by inherited human genetic variation. Previous studies have shown a strong effect of smoking on immune system. For example, cigarette smoke was shown to augment the production of numerous pro-inflammatory cytokines such as *TNF* (30, 31) and to decrease the levels of anti-inflammatory cytokines such as *IL-10* (32). Although previous study (33) and our study have not found a significant association between cigarette smoking and the risk of NHL, our joint interaction analysis of the genetic variants and smoking showed a significant association with the risk of NHL. This suggests that the effect of environmental factors, such as smoking, on the risk of NHL may be modified by single nucleotide variants, which may change immunoregulatory gene functions and therefore influence NHL risk through the immunoregulation pathways critical for lymphomagenesis.

This study features some limitations. One limitation is the sample size, which may influence the statistical power (34). Appropriately large sample size can provide precise estimation of unknown parameters. Small sample size may weaken the internal and external validity of study (35). The other limitation is that all participants (both cases and controls) are from the same hospital, which may influence our analysis and result. Further studies can be conducted using matched healthy individuals as controls. This study showed several significant associations between SNPs, environmental risk factors, gene and environment interaction, and risk of NHL. We need to replicate the results in other independent cohorts. In the future we can explore other methods when performing gene and environment interaction analysis. For example, we can try to use genome-wide association study to identify and include more genetic variants associated with NHL for the interaction analysis.

## Conclusions

Collectively, our analyses demonstrate that environmental variables hair dying and environmental tobacco smoking are significantly associated with the risk of NHL. Similarly, genetic factors rs1800893, rs4251961, rs1800630, rs13306698, and rs1799931 also show statistically significant association with the risk of NHL. We only observe that the interaction of smoking and weighted GRS has statistically significant association with NHL.

## Consent for Publication

Informed consent was obtained from all individual participants in the study.

## Data Availability

The individual level genetic and environmental data are available upon request.

## Author Contributions

JZ performed the data analysis and drafted the manuscript. XY and HF designed the data collection in the study. CW performed laboratory and field works. WX and PH supervised the study and revised the manuscript. All authors have reviewed and approved the manuscript.

### Conflict of Interest Statement

The authors declare that the research was conducted in the absence of any commercial or financial relationships that could be construed as a potential conflict of interest.

## Abbreviations

BH: Benjamini and Hochberg
BMI: body mass index
CI: confidence interval
DLBCL: diffuse large B-cell lymphoma
ERS: environment risk score
G×E: gene-environment
GRS: genetic risk score
GWAS: genome-wide association study
HWE: Hardy-Weinberg equilibrium
NHL: non-Hodgkin lymphoma
OR: odds ratio
RFLP: Restriction Fragment Length Polymorphism
SNP: single-nucleotide polymorphism.
