# Supplementary material for: Modeling Gene-Environment Interaction for the Risk of Non-hodgkin Lymphoma
Source: Front Oncol. 2019 Jan 14;8:657. doi: 10.3389/fonc.2018.00657 (PMC6340069; doi:10.3389/fonc.2018.00657)
Supplement: Supplementary file 1 [file Data_Sheet_1.docx]

**Supplementary**

**Supp Table 1: Interaction analysis between environmental variable and unweighted GRS**

| **Environmental Variable** | **ROR(95% CI) ^1^** | **P^2^** | **P_BH_^3^** | **ROR*(95% CI*) ^4^** | **P*^5^** | **P_BH_*^6^** |
| --- | --- | --- | --- | --- | --- | --- |
| Smoking | 0.41 ( 0.18 , 0.94 ) | 0.04 | 0.42 | 0.39 ( 0.1 , 1.42 ) | 0.15 | 0.70 |
| Alcohol | 0.88 ( 0.33 , 2.36 ) | 0.8 | 0.88 | 1.6 ( 0.33 , 7.71 ) | 0.56 | 0.70 |
| Hairdye | 2 ( 0.83 , 4.77 ) | 0.12 | 0.70 | 2.22 ( 0.59 , 8.37 ) | 0.24 | 0.70 |
| Farmexp | 0.8 ( 0.34 , 1.87 ) | 0.61 | 0.88 | 0.43 ( 0.12 , 1.58 ) | 0.21 | 0.70 |
| Environmental tobacco smoking | 0.82 ( 0.33 , 2.05 ) | 0.67 | 0.88 | 0.46 ( 0.11 , 1.81 ) | 0.26 | 0.70 |
| Benzene | 0.35 ( 0 , Inf ) | 1 | 1.00 | 0.51 ( 0 , Inf ) | 1 | 1.00 |
| Solvent | 0.52 ( 0.16 , 1.7 ) | 0.28 | 0.70 | 0.52 ( 0.07 , 3.66 ) | 0.51 | 0.70 |
| Metal | 2.56 ( 0.24 , 26.87 ) | 0.43 | 0.74 | 3.75 ( 0.16 , 89.62 ) | 0.41 | 0.70 |
| Agrichem | 0.51 ( 0.16 , 1.67 ) | 0.27 | 0.70 | 0.47 ( 0.08 , 2.72 ) | 0.4 | 0.70 |
| Others | 0.4 ( 0.05 , 2.97 ) | 0.37 | 0.74 | 0.5 ( 0.03 , 8.02 ) | 0.63 | 0.70 |
| Pesticide | 0.53 ( 0.16 , 1.74 ) | 0.29 | 0.70 | 0.46 ( 0.08 , 2.72 ) | 0.39 | 0.70 |

^1^ ROR, rate of odds ratio; 95% CI, 95% confidence interval. ^2^ P, p value of interaction analysis between binarized weighted GRS and environmental variables. ^3^ P_BH_ is the p value adjusted by BH method. ^4^ ROR*, rate of odds ratio after adjusting age, gender, education, family history of cancer and BMI; 95% CI*, 95% confidence interval after adjusting age, gender, education, family history of cancer and BMI. ^5^ P*, p value of interactive term using multiple logistic model with adjusting age, gender, education, family history of cancer and BMI. ^6^ P_BH_* is the p* value adjusted by BH method.

**Supp Table 2: Interaction analysis between individual ERS and GRS**

| Interaction analysis | ROR(95% CI) ^1^ | P^2^ | ROR*(95% CI*) ^3^ | P*^4^ |
| --- | --- | --- | --- | --- |
| riskScore^5^_Unweighted*ERS | 1.04 (0.83, 1.31) | 0.71 | 1.07(0.72, 1.59) | 0.73 |
| riskScore_Weighted*ERS | 1.49(0.94, 2.36) | 0.09 | 1.61(0.76, 3.41) | 0.21 |
| GRS^6^_U*ERS | 1.2(0.43, 3.34) | 0.73 | 1.48(0.28, 7.84) | 0.65 |
| GRS_W*ERS | 1.52(0.5,4.65) | 0.46 | 2.3(0.39,13.57) | 0.36 |

^1^ ROR, rate of odds ratio; 95% CI, 95% confidence interval. ^2^ P, p value of interactive term between ERS and GRS. ^3^ ROR*, rate of odds ratio after adjusting age, gender, education, family history of cancer and BMI.; 95% CI*, 95% confidence interval after after adjusting age, gender, education, family history of cancer and BMI. ^4^ P*, p value using multiple logistic model adjusted with age, gender, education, family history of cancer and BMI.  ^5^ riskScore, continuous genetic risk score. ^6^ GRS, binarized genetic risk score.

**
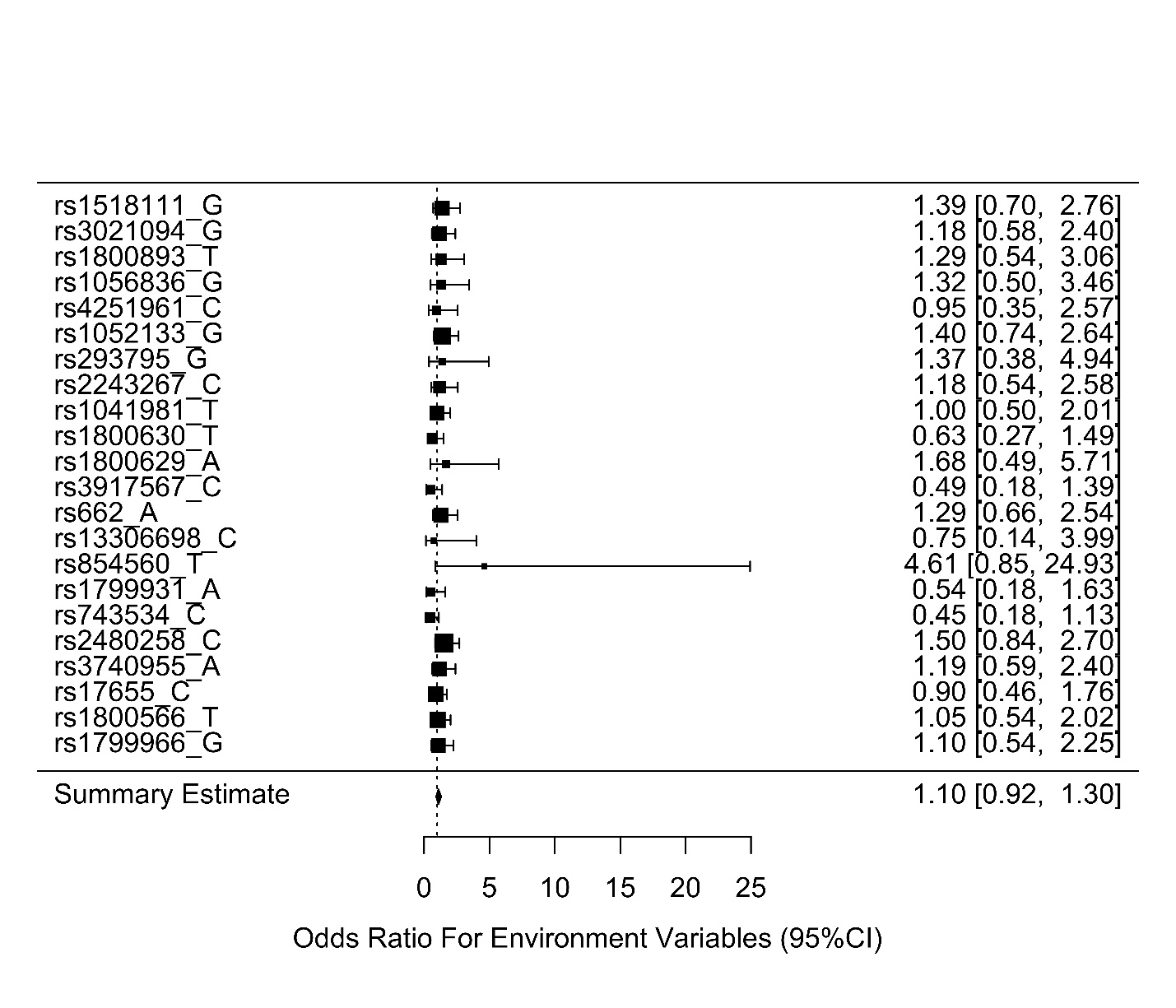
Supp Figure 1 Ratio of Odds ratios (95% confidence intervals) of risk of NHL**

This is based on the results from **Table 4**. Models are not adjusted for age, gender, education, family history of cancer and BMI.


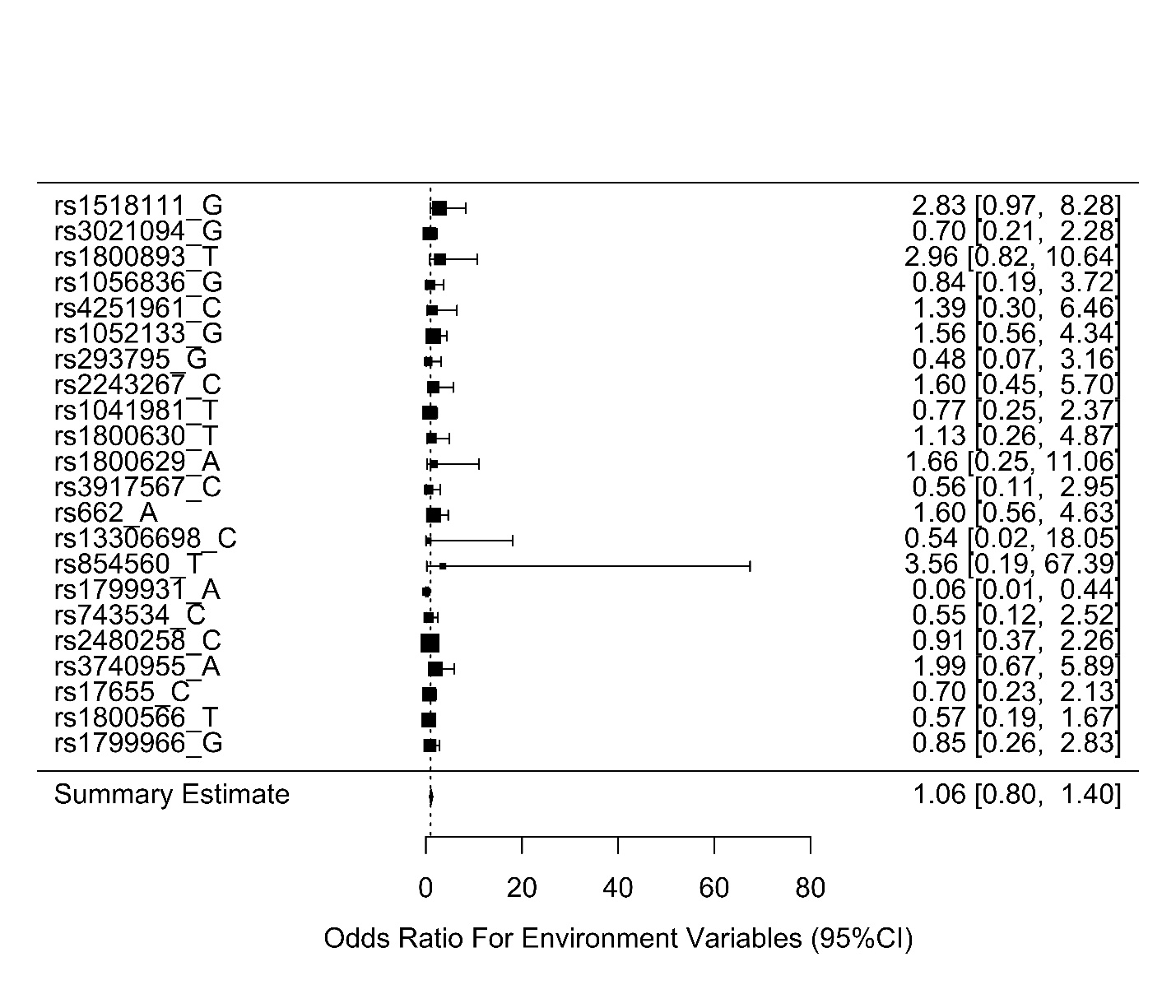


**Supp Figure 2 Ratio of Odds ratios (95% confidence intervals) of risk of NHL**

This is based on the results from **Table 4**. Models are adjusted for age, gender, education, family history of cancer and BMI.
